# Lasting effects of general anesthetics on the brain in the young and elderly: “mixed picture” of neurotoxicity, neuroprotection and cognitive impairment

**DOI:** 10.1007/s00540-019-02623-7

**Published:** 2019-03-11

**Authors:** Lingzhi Wu, Hailin Zhao, Hao Weng, Daqing Ma

**Affiliations:** 1 Anaesthetics, Pain Medicine and Intensive Care, Department of Surgery and Cancer, Faculty of Medicine, Imperial College London, Chelsea and Westminster Hospital, London, UK; 2 Department of Anesthesiology, Shanghai Fengxian District Central Hospital, Shanghai Jiao Tong University Affiliated Sixth People’s Hospital South Campus, Fengxian District, Shanghai, China

**Keywords:** General anesthetics, Brain, Neurotoxicity, Neuroprotection

## Abstract

General anesthetics are commonly used in major surgery. To achieve the depth of anesthesia for surgery, patients are being subjected to a variety of general anesthetics, alone or in combination. It has been long held an illusory concept that the general anesthesia is entirely reversible and that the central nervous system is returned to its pristine state once the anesthetic agent is eliminated from the active site. However, studies indicate that perturbation of the normal functioning of these targets may result in long-lasting desirable or undesirable effects. This review focuses on the impact of general anesthetic exposure to the brain and summarizes the molecular and cellular mechanisms by which general anesthetics may induce long-lasting undesirable effects when exposed at the developing stage of the brain. The vulnerability of aging brain to general anesthetics, specifically in the context of cognitive disorders and Alzheimer’s disease pathogeneses are also discussed. Moreover, we will review emerging evidence regarding the neuroprotective property of xenon and anesthetic adjuvant dexmedetomidine in the immature and mature brains. In conclusion, “mixed picture” effects of general anesthetics should be well acknowledged and should be implemented into daily clinical practice for better patient outcome.

## Introduction

Modern anesthesia enabled increasingly complicated surgical and diagnostic procedures to be performed safely on patients, and has significantly advanced human medicine. For years after its advent, it was believed that general anesthetics (GAs) exert reversible, temporary effect on the central nervous system, which would return to its pristine state once the anesthetic exposure is ceased. The long-lasting effects including cellular signaling changes and their impact after anesthetic exposure are enormous [1]. These effects can be desirable or undesirable. Indeed, anesthetics received during surgery were shown to be associated with brain dysfunction in young and elderly [2, 3]. In years to come, a large body of pre-clinical studies, and accumulating clinical evidences has steadily strengthened the belief that anesthetics may produce morphological changes and long-term functional impairment in brains at the extremes of age. Amidst the growing evidences linking GAs to neurocognitive impairment, the United States Food and Drug Administration issued a precautionary communication on GA use in patients aged three years and under [4], accentuating GA-related public health concerns. In this review, we attempt to provide a comprehensive discussion on the unwanted effects of general anesthetics on the central nervous system (CNS), integrating pre-clinical findings with clinical evidences.

Mechanism studies revealed that GAs act through various receptor proteins to modulate neuronal activities, to exert their amnesic, analgesic, sedative and immobilizing effects. The most recognized receptor targets include GABA_A_ receptor (propofol, etomidate, isoflurane, sevoflurane), NMDA receptor (nitrous oxide, xenon, ketamine), glycine receptor and two-pore potassium channel [5, 6]. Such inhibitory and activating receptors are abundant throughout the mammalian brain, and may mediate unwanted, off-target effect of GAs to precipitate long-term cognitive dysfunction. In this regard, the extraordinary plasticity/connectivity and reduced compensating capacity of the developing and aging brains, respectively, may make them vulnerable to the ubiquitous, undesired actions of general anesthetics.

## The developing brain

### General anesthetics and neurotoxicity

Over the years, cellular and animal studies yielded substantial and convincing evidence on the cytotoxic and neurotoxic properties of general anesthetics. Since the pioneering study by Jevtovic-Todorovic et al., whereby 6 h exposure to a mixture of nitrous oxide, isoflurane and midazolam in postnatal day 7 rats induced long-term learning deficits [7], studies have demonstrated that routine GAs (isoflurane [8, 9], sevoflurane, propofol [10, 11], ketamine [12]) are capable of producing lasting cognitive, behavioral and memory deficiency in rodents when exposed in the early postnatal period. Studies on non-human primates mirrored such findings, wherein early-life exposure to ketamine, sevoflurane or isoflurane led to persistent decline in cognitive, executive, memory and motivation-based tasks, and increased anxiety behaviors in the long term [13–15]. Based on the cumulative findings, the potency of GAs on neurobehavioral development is likely determined by the total length of exposure (a single lengthy vs. repeated brief exposures) and the developmental stage of the animal (first week postnatal). It was also reported that age of the neuron per se better predicts vulnerability to GAs than age of the organism, wherein juvenile neurons in adult animals are susceptible to the effect of GAs [16], to suggest neurocognitive toxicity of GAs even in adulthood.

**Fig. 1:**
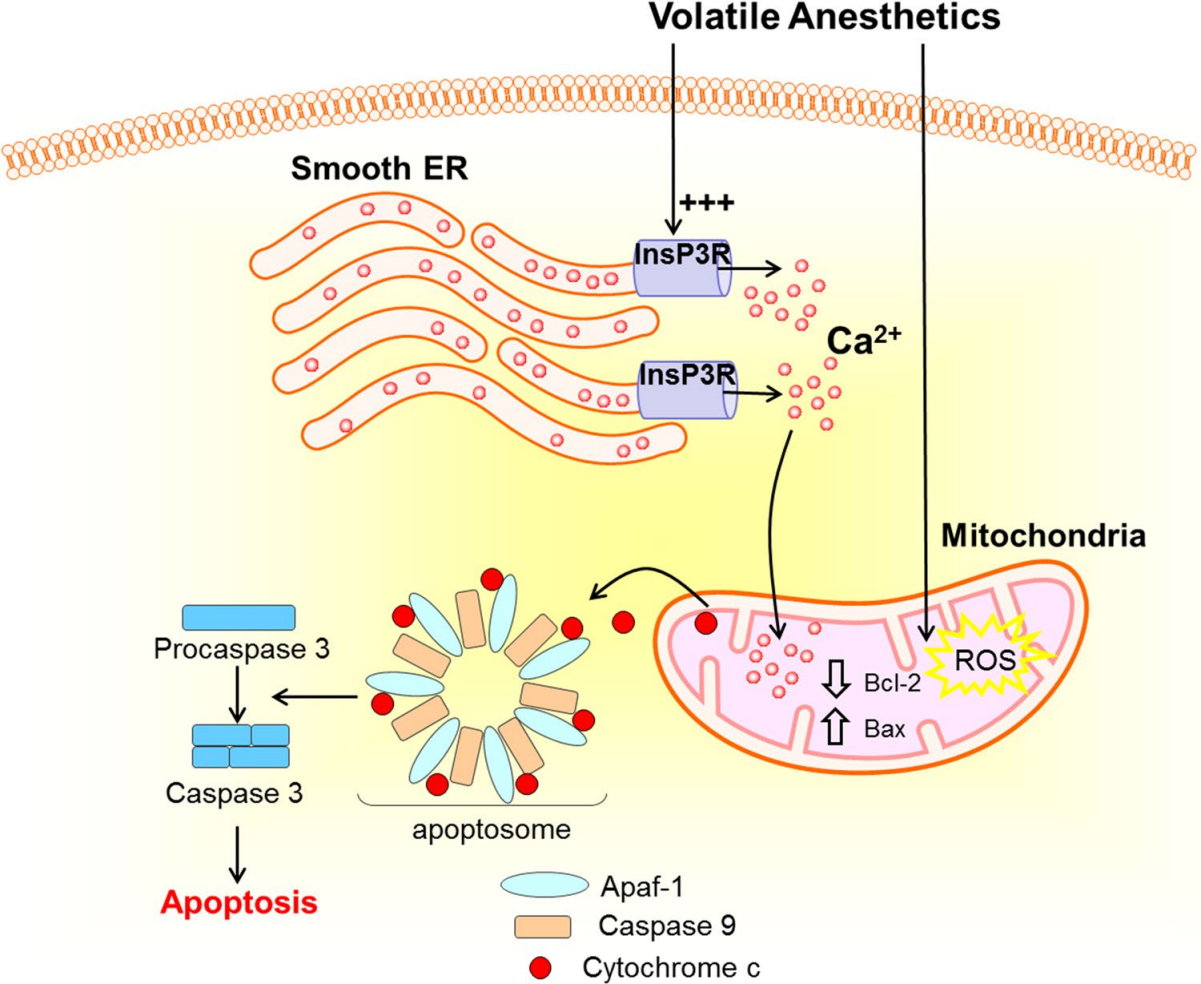
Neurotoxicity and volatile anesthetics. Volatile anesthetics were shown to activate mitochondrial apoptosis pathway, by increasing mitochondrial ROS production, lowering anti-apoptotic Bcl-2/pro-apoptotic Bax ratio and promoting cytochrome C from mitochondrion into cytosol to form apoptosome, which subsequently cleaves pro-caspase 3 to caspase 3. In addition, volatile anesthetic isoflurane was demonstrated to directly activate and open inositol 1,4,5-trisphosphate receptor (InsP3R) calcium channel located on the smooth endoplasmic reticulum. Excessive opening of InsP3R calcium channel by isoflurane leads to significant Ca2 + leakage from ER and cause mitochondrial Ca2 + overload, which could aggravate cytochrome C release and caspase cleavage pathway. *Apaf-1* Apoptotic protease-activating factor 1, *Bax* Bcl-2-associated X protein, *Bcl-2* B-cell lymphoma 2 protein, *Ca*^*2+*^ calcium ion, *InsP3R* inositol 1,4,5-triphosphate receptor, *ROS* reactive oxygen species

The molecular mechanisms underlying GA’s lethality in developing neurons have been extensively explored. In vitro studies consistently reported the role of mitochondria and intrinsic (mitochondrial) apoptosis in GA-induced neurotoxicity. In neuronal culture and brain slice derived from immature rodents, isoflurane exposure significantly decreased anti-apoptotic BCL-2/pro-apoptotic Bax ratio, increased reactive oxygen species (ROS), and promoted cytochrome C release from mitochondria and caspase 3 cleavage [17–19]. Subsequent studies identified inositol 1,4,5-trisphosphate receptor (InsP3R) located on endoplasmic reticulum (ER) as a novel target of GA and an upstream signaling component of mitochondria. Under physiological conditions, activation of the InsP3R leads to Ca^2+^ release from ER lumen into the cytosol to initiate calcium-dependent signaling. Isoflurane was shown to directly open InsP3R channels to induce excessive Ca^2+^ release from ER into cytosol and mitochondria, which further leads to mitochondrial calcium overload, ATP production failure, cytochrome C release and caspase activation [20–22]. In addition to targeting the mitochondria, recent data suggests that GA-induced cytosolic calcium buildup also impairs autophagosomal and autolysosome function to reduce cytoprotective autophagy, which would bias cell towards apoptosis [23] (Fig. 1).

Retrospective cohort studies found that multiple rounds of anesthetic exposure, and in young children under 2–4 years of age, were associated with learning difficulty and academic underachievement during childhood and adolescence [24, 25]. Single, brief anesthetics exposure, on the other hand, in pediatric patients younger than 3 years of age, was not found to be associated with neurocognitive or behavioral impairment [26]. However, one study reported that both single and multiple exposures to anesthesia were linked to language and abstract reasoning deficits [27]. The discrepancy is likely due to the selection bias inherent to retrospective study design, different assessment parameters, and/or age at assessment. Two prospective clinical studies examined the effect of single general anesthetic exposure at young age on future neurocognitive performance. The General Anesthesia compared to Spinal anesthesia (GAS) trial showed that GA is not associated with cognitive impairment compared to awake SA at 2 years of age [28]. The Pediatric Anesthesia Neurodevelopment Assessment (PANDA) trial also did not observe significant decline in cognitive, behavioral and memory capacity in GA-exposed subjects in comparison to their unexposed siblings, at 8–15 years of age [29]. Nevertheless, such findings cannot rule out the possibility that longer duration, repeated anesthetic exposure can harm the developing brain.

These studies are present with various confounding factors that warrant cautious interpretation of results. As anesthetics are rarely given alone, these studies rather assessed the association between surgery plus anesthetic exposure and cognitive/behavioral deficiency, instead the risks associated with anesthetics per se [30, 31]. In this regard, it would be difficult to dissect out the effect of surgery on neurocognitive development; moreover, children requiring surgery at young age are known to be different in many ways from those who do not, and such developmental differences may contribute to neurocognitive deficit attributed to surgery and/or anesthesia. Furthermore, confounders such as hypotension, body temperature, and hypoxia during surgery are rarely described/controlled for in these studies, and could potentially alter the outcomes. In view of such, it would be very difficult to establish whether general anesthetics are causally linked to cognitive and behavioral deficiency, or conditions associated with such. Thus, large-scale observational studies and randomized trials with longer duration exposure of GAs and follow-up, more sensitive outcome measures, and stringent confounder control are required in the future, to provide more conclusive and informative data.

### Neuroprotection in hypoxic-ischemic brain injury

Cerebral hypoxic brain injury contributes significantly to perinatal mortality and morbidity worldwide. It affects approximately 4 in 1000 births [32] and causes permanent neurological deficits in 25% of sufferers [33]. It is estimated that 4 million babies die in the neonatal period every year and birth asphyxia accounts for 23% of these deaths [34]. The lifelong consequences of perinatal hypoxic-ischemic encephalopathy to the affected infants, their family and the society necessitate the development of novel neuroprotective strategies. Hypoxic brain injury develops when oxygenation of the brain tissue is reduced, usually due to cardiac arrest or cerebrovascular incidents [35]. In the adult brain, this mostly occurs in the form of stroke. In infants, the most common type of hypoxic brain injury is due to ischemia superimposed on hypoxia [33]. During or after birth, reduction in cerebral blood flow or further deoxygenation of the blood leads to the pathological asphyxia. The leading cause of hypoxic brain injury in the newborn is placental blood flow abruption and impaired gas exchange [36]. The brain injury is diffuse not focal, and affects the whole brain homogeneously [33]. During hypoxia/ischemia brain injury, energy depletion is due to the hypoxemia that switches cellular metabolism from aerobic to anaerobic. Anaerobic metabolism is insufficient to meet the cellular energy demands, which lead to depletion of stored ATP, creatinine phosphate and other forms of energy [37, 38]. Basic cellular proteins such as the Na^+^/K^+^-ATPase no longer function properly, leading to Na^+^ and Ca^2+^ influx, followed by cytotoxic edema and lysis [38, 39]. The brain tissue of the affected areas has a biphasic response to a hypoxic-ischemic injury [40, 41]. First, there is primary cell death, which includes the death of affected cells via necrosis during or shortly after the hypoxia, then secondary cell death via apoptosis 8–72 h after the hypoxia [42] or through autophagosomal or lysosomal death [43].

Glutamate neurotoxicity, or excitotoxicity, is the overstimulation of neuronal cells by glutamate that is released due to the depolarized membrane, and is a central feature to hypoxic-ischemic brain injury. Ischemic insult causes significant release of glutamate from excitatory nerve terminals, to promote water influx via the opening of surface channels such as the AMPA receptors and further influx of Ca^2+^ through the NMDA receptors on post-synaptic neuron membrane [44]. Accumulation of cytosolic Ca^2+^ leads to free radical production through formation of xanthenes and prostaglandins, cell membrane damage, phospholipase C activation, activation of endonucleases as well as apoptosis proteins, ultimately leading to cell death [45].

**Fig. 2:**
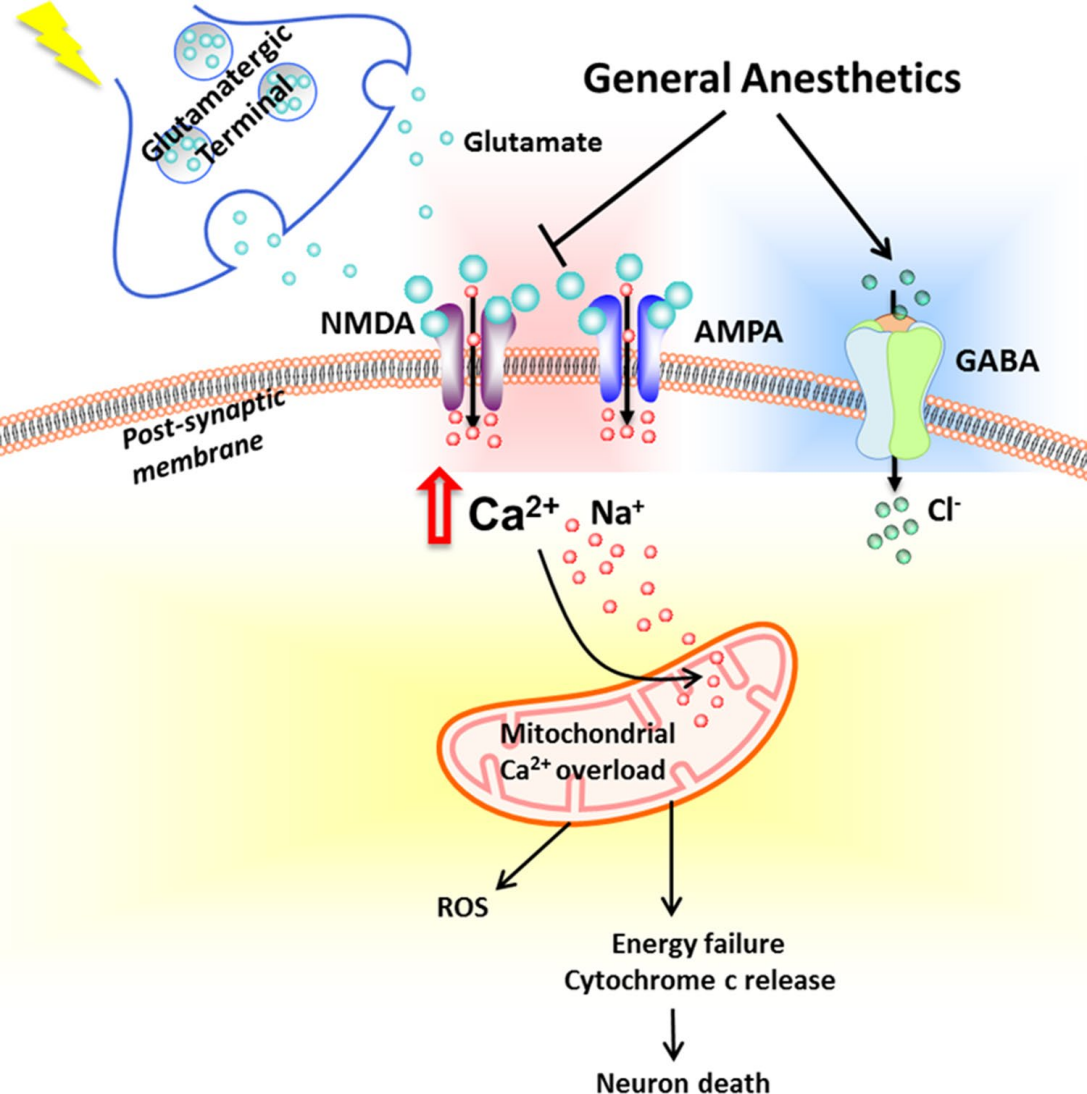
Excitotoxicity and general anesthetics. Glutamate released from pre-synaptic nerve terminals bind to NMDA and AMPA receptors on the post-synaptic membrane to lead to calcium ion (Ca^2+^) influx and membrane depolarization. Excessive glutamatergic signaling and calcium accumulation would result in mitochondrial calcium overload, reactive oxygen species (ROS) production, cellular energy failure, apoptosis protein (cytochrome C) release/activation, and ultimately neuron death. Activation of GABA receptor leads to chloride ion (Cl^−^) influx to hyperpolarize membrane and thus inhibits depolarization. Volatile anesthetics (in particular isoflurane) have been shown to antagonize NMDA and AMPA, inhibit Ca^2+^ influx and protect neuron death from ischemia-induced excitotoxicity. Isoflurane also agonizes GABA receptor to hinder excitatory neurotransmission. *AMPA* a-amino-3-hydroxyl-5-methyl-4-isoxazole-propionate, *Ca*^*2+*^ calcium ion, *Cl*^*−*^ chloride ion, *GABA* gamma-aminobutyric acid-A, *Na*^*+*^ sodium ion, *NMDA N*-Methyl-d-aspartic acid, *ROS* reactive oxygen species

Much work has focused on the excitotoxicity-antagonizing effect of general anesthetic. Early studies reported that isoflurane directly interacts with the glutamatergic N-Methyl-D-aspartic acid (NMDA) receptor, whereby isoflurane may suppress NMDA or α-amino-3-hydroxyl-5-methyl-4-isoxazole-propionate (AMPA)-induced glutamate release, calcium uptake, mitochondrial membrane depolarization and excitatory neurotransmission [46–48]. Inhalational anesthetics sevoflurane and halothane were also shown to inhibit NMDA-induced excitotoxicity and calcium transient, however, the extent of neuroprotection appears to be lesser than that of isoflurane [48]. In this regard, excitotoxicity antagonism has been regarded as one of the mechanisms inhalational anesthetics act through to protect against ischemic brain injury. In addition, isoflurane interacts with and agonize inhibitory gamma-Aminobutyric acid-A (GABA) receptor that would inhibit depolarization and excitatory neurotransmission [49, 50] (Fig. 2).

Similar to that of volatile anesthetics isoflurane and nitrous oxide, xenon inhibits the plasma membrane Ca^2+^ pump, which might be responsible for neuronal Ca^2+^ concentration increase and altered excitability [51]. In 1998, it was shown that xenon suppresses nociceptive responsiveness through inhibition of NMDA receptors [52]. Xenon is different from all other volatile anesthetic agents, as it exerts no action on GABA_A_ receptors [53]. It was predicted through biochemical modeling that xenon binds at the glycine site of the NMDA receptor and causes potent non-competitive inhibition [54]. Compared with commonly used anesthetic agents, xenon-induced anesthesia is featured with greater circulatory stability, lower analgesic consumption, lower adrenergic levels and better perfusion of individual organs [55]. Furthermore, xenon’s anesthetic effect is 1.5 times greater than that of nitrous oxide [56]. Nowadays, xenon has been used in anesthesia for many different types of surgery [57, 58].

Following the discovery that xenon is capable of inhibiting NMDA receptors, it was naturally postulated that xenon can protect neuronal cell against injury, since NMDA receptor-mediated neurotoxicity plays a critical role in neuronal cells death. Ma et al. [59] demonstrated the neuroprotective effect of xenon through N-methyl (D, L)-aspartate-induced neurotoxicity. Later, Ma et al. [60] demonstrated that xenon preconditioning improved both morphology and neurological functional outcome after the hypoxia–ischemia insults. The mechanism of xenon preconditioning may be due to increased synthesis of survival proteins such as Bcl-2.The effect of xenon-mediated organoprotection was investigated in combination with methods in general clinical practice. Ma et al. [61] demonstrated that combination of xenon and hypothermia caused a synergistic enhancement of their individual neuroprotective properties. In addition to preconditioning, there are also studies demonstrating the effectiveness of post-treatment of xenon in brain injury. Dingley et al. [62] showed that xenon administered after a hypoxic-ischemic insult in neonatal rat model conferred uniformly 80% neuroprotection, as assessed by neuropathology of the major areas of the brain. The neuroprotective effect of xenon was further tested on large animals. Schmidt et al. [63] evaluated whether xenon provides a neuroprotective effect to attenuate brain injury after transient cerebral ischaemia due to cardiac arrest in pigs. The major findings were that during reperfusion, brain injury is significantly smaller with the xenon treatment than control. Faulkner et al. [64] compared the effect of hypothermia and xenon-augmented hypothermia on the brain after transient global hypoxia-ischemia in piglet, whereby combination with xenon further reduced levels of cell death and tissue damage. In 2010, xenon exposure combined with hypothermia was conducted for the first time on a newborn baby suffering from hypoxia at birth. The treatment was demonstrated to be very effective in attenuating mild brain injury in the young [65].

**Fig. 3:**
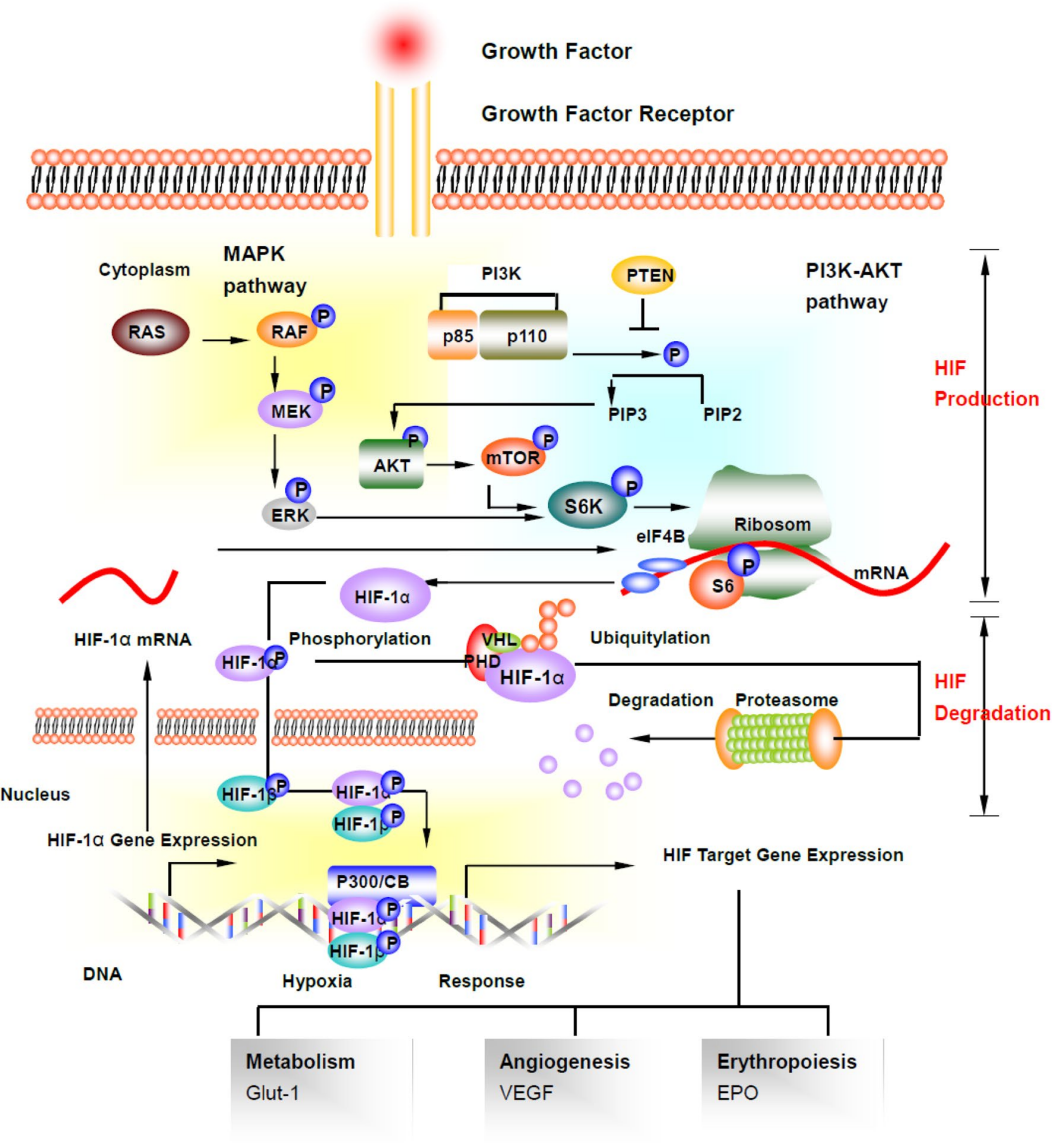
The hypoxia-inducible factor-1 (HIF-1) signaling pathway. Volatile anesthetics has been shown to activate or suppress HIF-1 system. HIF-1 is a heterodimer that consists of HIF-1α (120 kDa) and HIF-1β (91–94 kDa), HIF1β is expressed constitutively in all cells and remains stable regardless of oxygen tension. At normoxia conditions, HIF-1α combines with the tumor-suppressor Von Hippel–Lindau (VHL) protein through a hydroxylated proline residue and is then hydroxylated by prolyl-4-hydroxylases (PHD) in the cytoplasm, and this interaction causes HIF-1α to be ubiquitinated and to be targeted by proteasome-mediated protein degradation. Under hypoxic conditions, oxygen deficiency inhibits the activity of prolyl hydroxylases and leads to the accumulation of HIF-1α. Production of HIF-1α is controlled by PI-3K/AKT/mTOR pathway and partially influenced by MAPK pathway, phophorylation of AKT and mTOR leads to translation of HIF-1α. HIF-1α is translocated into the cell nuclear and together with HIF-1β bind to hypoxia-response elements (HREs). A broad range of protective pathways is activated, which regulate several aspects of cellular activities, such as angiogenesis, erythropoiesis, cell proliferation, cell survival and energy metabolism. *AKT* protein kinase B, *HIF-1* hypoxia-inducible factor-1, *MAPK* mitogen-activated protein kinase, *mTOR* mammalian target of rapamycin, *PI-3K* phosphatidylinositide 3-kinases

Hypoxia-inducible factor-1 (HIF-1) is the central mediator of the cellular response to hypoxic environments, and represents a key mechanism that inhalational anesthetics act upon to provide neuroprotection against ischaemic brain injury [66]. HIF-1 is a transcription factor belonging to the basic helix-loop-helix–Per-Arnt-Sim (bHLH–PAS) family. It is a heterodimer composed of α and β subunits; α subunit is continuously made and degraded in both normoxic and hypoxic conditions, whilst β subunit is insensitive to oxygen [67]. HIF-1 mutations are known to lead to neural tube defects, brain underdevelopment and decreased neuronal cell number [68]. HIF-1 can be activated during hypoxia due to accumulation of the α subunit through reduced degradation [67]. The degradation of the HIF-1α subunit is mediated through the Von Hippel–Lindau (VHL) tumor-suppressor protein. VHL interacts with Elongin C and ubiquitinates HIF-1α, targeting it for proteosomal degradation [67]. HIF-1 hydroxylation is promoted by propyl hydroxylase (PHD), which is oxygen dependant [69]. Growth factors such as insulin-like growth factor (IGF)-1 and FGF bind to their receptors and activate the PI3 kinase/Akt/mTOR pathway, initiating HIF-1α production [70]. The critical gene expression after HIF activation mediates cellular responses to the hypoxia, including enhanced cell survival, erythropoiesis and angiogenesis [68] (Fig. 3). In this regard, volatile anesthetic halothane was shown to inhibit hypoxia-induced activation of HIF-1 [71], whereas isoflurane and xenon were demonstrated to upregulate HIF-1α expression and activity to confer kidney protection against ischemic insult [72–75]. Similarly, neuroprotection by isoflurane and xenon in ischemic brain injury is accompanied by HIF-1a upregulation [76, 77], which likely owes to volatile anesthetics’ ability to activate PI3K/AKT and ERK1/2 phosphorylation pathways.

## Elderly and cognition

### Cognitive disorders

Cognitive disturbance is commonly observed in elderly patients following surgery and general anesthesia and is predictive of short- and long-term outcomes. Cognitive disorders include postoperative delirium (POD) and postoperative cognitive dysfunction (POCD). Unlike POD, as of current, accurate diagnosis of POCD is made difficult by the lack of formal, universal diagnosis criteria and the varying degree/extent of symptom presentation. POD is an acute and transient disturbance that affects early postoperative period (days to weeks), whereas POCD manifests as more subtle deteriorations in memory, attention, and cognition over a much longer period of time (months to years). The observed incidence of POD ranges from 10 to 45% that increases with age and surgery complexity/risk [78], and POCD was reported to affect 26% patients over 60 years in the first postoperative week, and the incidence falls to 10% in the following 3 months [79]. However, clinical evidence attributing POCD to surgery and anesthesia exposure are inconclusive [80], as long-term follow-up study found only 1% of elderly subjects suffer from persistent POCD 1–2 years onwards [81], with pre-operative cognitive performance (e.g., mild cognitive decline, possible/probable Alzheimer’s disease) being a better predictor of postoperative cognitive trajectory [82]. The general consensus now is that POCD is multi-factorial with various biological and socioeconomic predisposing factors. Despite the debatable cause of POCD syndromes, it remains a perioperative priority that demands all plausible factors to be considered for lowering its incidence. Amongst the many predisposing factors, the choice of anesthetic agent/technique represents a likely one [83, 84].

Clinical studies investigating the potential role of anesthetics in POCD have been inconclusive. When compared with regional anesthesia, general anesthesia was not found to be associated with significantly higher incidence of delirium and POCD in elderly patients 3 months following surgery [85, 86]. In another study, incidence of POCD in the first postoperative week was shown to be independent of the mode of anesthesia (light sedation, intravenous or inhalational) and the type of surgery [87]. In two studies that examined the effect of anesthesia depth (as determined by BIS index) on cognitive function in elderly, one found that deep anesthesia (BIS < 20) was associated with higher incidence of delirium but not POCD [88], whereas the other showed that light anesthesia (BIS 55–70) impaired information processing ability 4–6 weeks after surgery [89]. Moreover, a case–control study examining later life POCD risk reported that GA exposure after age of 40 was not associated with increased rate of mild cognitive impairment in subjects of 70–89 years of age [90]. A randomized trial reported that sevoflurane was associated with better postoperative cognitive function than propofol-based anesthesia following on-pump cardiac surgery, and it was suggested that sevoflurane could compensate for the cognitive consequences from intraoperative cerebral desaturation [91]. In these clinical scenarios, it should be noted that the effect of surgery or any co-morbidities is likely to be more prominent and could have masked the subtle influences of anesthesia on cognitive performance. Clinical observations could be further confounded by the lack of reliable diagnostic criteria and timing for POCD, as mentioned above.

A large number of animal studies have been conducted to examine the deleterious effect of general anesthesia in the absence of surgical insults. Volatile anesthetics isoflurane [92] and desflurane [93] were found to induce long-term impairment in spatial memory acquisition and learning in 18- to 20-month-old rats, when assessed by radial arm maze. Such findings were challenged by another study that demonstrated isoflurane exposure did not impair spatial memory and learning (Morris Water Maze) in aged rats, which corresponded to isoflurane’s lack of effect on neurogenesis and cell death within the hippocampus [94]. Other general anesthetics were also reported to lack detrimental effects. Sevoflurane did not impair acquisition learning and memory in 20- to 24-month-old rats and may even improve long-term learning capacity [95]. Similarly, long-term spatial memory and learning was also preserved in 18-month-old rats receiving intravenous propofol [96]. The collective findings suggest that GA choice could differentially affect long-term cognitive outcome, with certain anesthetics being more favorable in susceptible elderly patients. To answer such questions, studies comparing the cognitive effects of different anesthetics at equipotent doses and of comparable exposure durations would be required. A recent clinical study indicated that in comparison with sevoflurane, propofol-based general anesthesia decreased the incidence of delayed neurocognitive recovery, a derivative form of POCD, in older adults after major cancer surgery [97].

In the search of prophylaxis and therapy against POD, the anesthetic adjuvant dexmedetomidine has been shortlisted as a promising candidate. As an alpha_2_-adrenergic receptor agonist, it is hypothesized that dexmedetomidine interacts with different physiological and biochemical pathways within the CNS to achieve multitude anti-delirium neuroprotection. Dexmedetomidine binds to a_2_ adrenoceptors in locus ceruleus (LC) to inhibit neuronal activity within LC, which subsequently leads to release of inhibitory neurotransmitters GABA and galanin into the cortex to promote natural sleep-like sedation [98, 99]. Moreover, dexmedetomidine also reduces the requirement of benzodiazepines and opioids throughout the perioperative period, and this could thus reduce delirium occurrence due to benzodiazepine/opioid use. Using animal models of surgical trauma and/or anesthesia exposure, it has been demonstrated that Dex has anti-apoptosis [100] and anti-inflammatory [101] properties that is associated with improved neurocognitive outcome. We proceeded to test the delirium-attenuating potential of dexmedetomidine in a prospective randomized trial, which enrolled 700 elderly patients to receive low-dose dexmedetomidine or saline for overnight hours in ICU after non-cardiac surgery. The trial demonstrated that low-dose, prophylactic dexmedetomidine in patients > 65 years of age significantly reduced the incidence of postoperative delirium in the first week after surgery [102]. In the 3-year follow-up study of the trial, we further demonstrated that dexmedetomidine recipients showed significantly improved quality of life, cognitive function and long-term survival [103]. In a parallel, in a separate randomized trial study, we demonstrated that prophylactic dexmedetomidine in non-cardiac surgery patients increased non-rapid eye movement sleep and improved overall sleep quality, which likely contribute to the lowered incidence of delirium [104]. In an independent study, as opposed to prolonged infusion, Deiner et al. showed that dexmedetomidine administration at a relative high dose during the intraoperative period and 2 subsequent hours in non-cardiac surgery patients did not significantly reduce the occurrence of delirium in the first perioperative week and cognitive dysfunction at 3–6 months after surgery [105]. The collective findings highlight the short-acting nature of dexmedetomidine, and the need for continuous infusion and specific timing when using dexmedetomidine for delirium and POCD prevention and may be also patient population specific.

### Alzheimer’s disease

A number of pre-clinical studies on transgenic animals carrying AD-predisposing genetic alterations have examined the molecular, morphological and behavioral outcomes following exposure to general anesthetics. In these studies, the transgenic animals harbor one or more genetic mutations of the amyloid protein cascade, including mutation to amyloid precursor protein (APP) and/or proteolytic enzyme that cleaves APP (e.g., γ-secretase—presenilin 1 or presenilin 2 mutation), which leads to excessive production of neurotoxic Aβ42 fragment and deposition of amyloid plaques. Additional mutation to tau protein can also be introduced to generate tri-transgenic animals that exhibit hyper-phosphorylated tau and neurofibrillary tangles (NFT), which better recapitulate the broad spectrum of AD pathology in human [106].

An early study demonstrated that intermittent isoflurane or halothane exposure for 5 days impaired cognitive function in 12-month-old wildtype but not Tg2576 transgenic mice (overexpressing mutant human APP), with halothane exposure significantly increasing amyloid deposition in Tg2576 subjects [107]. A plausible explanation could be that Tg2576 mice already exhibit significantly lower baseline cognition that does not deteriorate further upon anesthetic challenge, unlike wildtype animals. Another group found that repeated, prolonged isoflurane exposure over a course of 3 months hindered Y-maze ambulatory behavior in transgenic Tg2576 mice; isoflurane elevated Aβ_1–42_ amyloid deposition not only in transgenic animals but also in wildtype counterparts, despite such effects being more prominent in the transgenic group [108]. Thus, with the same genetic background, the neurotoxic effect of inhalational anesthetics is duration dependent and specific to different test paradigms, and a certain threshold of molecular alternation must be exceeded to produce noticeable cognitive regression. In APP695 mice that displays significant amyloidopathy in the hippocampus, 4 h exposure to 1MAC isoflurane significantly impaired spatial memory performance as assessed by Morris Water Maze [109], with the effects being more subtle in wildtype animals. In an attempt to study the combined effect of anesthesia plus surgery, 2 h exposure to isoflurane, but not desflurane, with brief laparoscopy significantly increased escape latency in Barne maze test in 5-month 5XFAD mice (transgenic with five familial Alzheimer’s disease mutations, amyloid deposition and intraneuronal Aβ_42_ accumulation are evident at only 2 months of age), indicating worsened cognitive function [110]. The same group further reported that sevoflurane with brief laparoscopy reduced freezing time of fear conditioning system in only female but not male 5XFAD mice, to suggest sex-dependent impairment to hippocampus-mediated cognitive function [111]. In other AD mouse models, the detrimental effect of inhalational anesthetics on cognition is less conclusive. Contrary to the previous neurotoxic findings, one study reported that 2 h of 1MAC isoflurane exposure significantly improved learning and cognition in 4-month-old APP23 mice (phenotypes include cerebrovascular amyloid deposition and mild neuronal loss), as well as wildtype mice [112]; the same isoflurane regime was not found to affect cognitive function in 14–16-month-old APP23 mice. In line with this, repeated exposure to 1MAC isoflurane over 4 weeks was not found to impair spatial working and cognition in tri-transgenic AD mice carrying APP, presenilin 1 and tau protein mutations, despite increased phosphorylated-tau expression in the hippocampus [113]. Collectively, the discrepant findings between different AD models do not constitute convincing evidences to support that GA exposure predisposes/accelerates Alzheimer’s disease.

There has been significant research effort to uncover the cellular and molecular mechanisms that could underlie general anesthetics’ potential neurotoxic effects on AD brain. Majority of studies focused on anesthetics’ ability to induce neuronal apoptosis and to potentiate AD-associated pathology, including defective amyloid protein pathway, tau protein hyperphosphorylation and neuroinflammation.

**Fig. 4:**
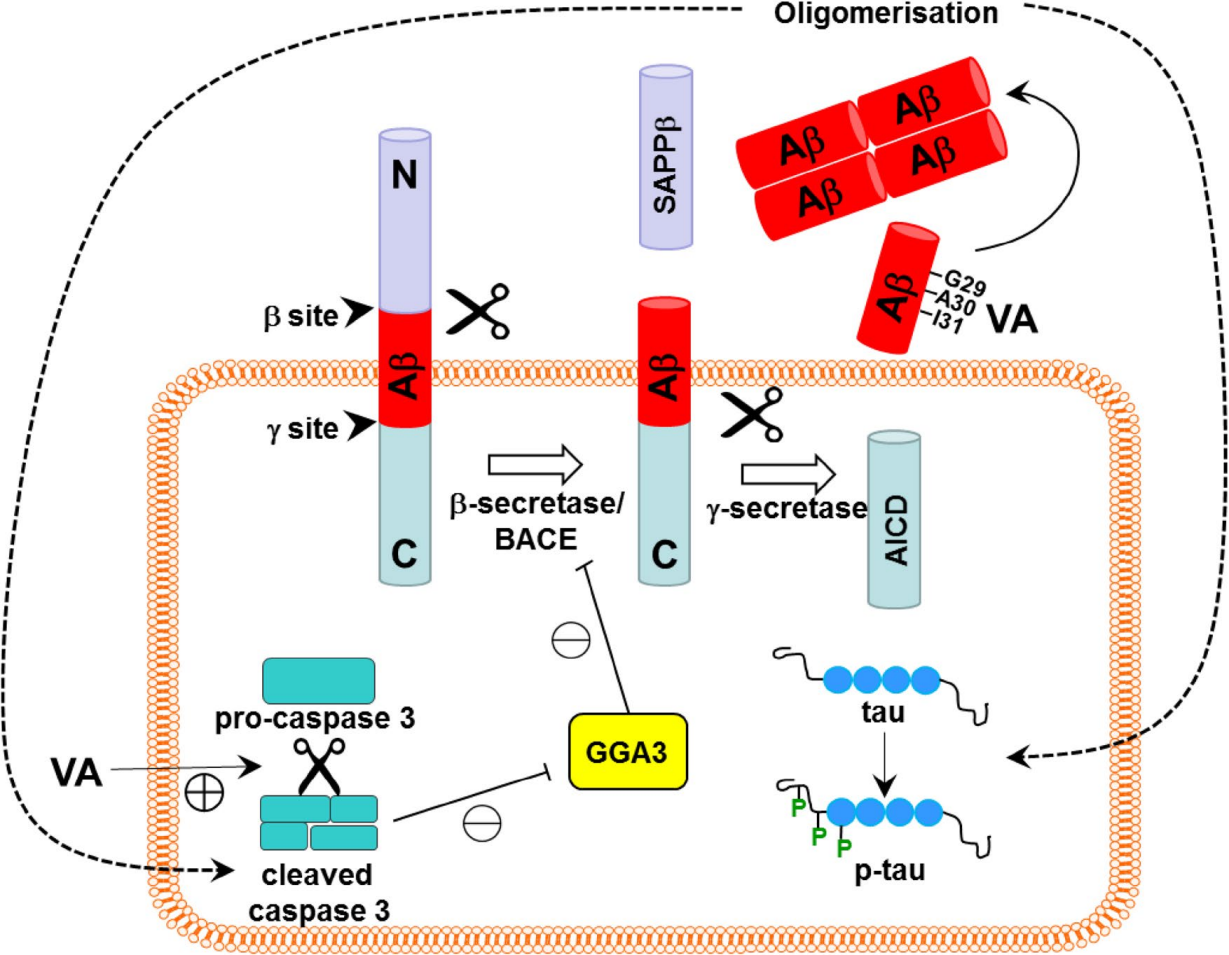
The hypothesized pathways of volatile anesthetics induced neuropathology associated with Alzheimer’s disease. Volatile anesthetic was demonstrated to promote toxic Ab production and aggregation, and such effect was shown to be downstream of VA-induced caspase-3 cleavage and activation. The cleaved caspase 3 could inactivate Golgi-associated, gamma adaptin ear-containing, ARF-binding protein 3 (GGA3), which degrades b-site APP-cleaving enzyme (BACE, or b-secretase). The overall effect is cellular stabilization of BACE/b-secretase and increased processing of amyloid progenitor protein (APP) by β-secretase and γ-secretase, leading to accumulation of neurotoxic Ab aggregates. Reciprocal regulation of this pathway may also exist, whereby inhibiting Ab oligomerization could reduce caspase 3 activation. Caspase 3 activation and Ab accumulation could also be upstream of tau phosphorylation, as Ab oligomerization inhibitor prevented tau hyper-phosphorylation. In addition, owing to their small molecular size, volatile anesthetics have been shown to directly interact with residues G29, A30 and I31 of Ab to promote Ab oligomerization. *Ab* amyloid-beta protein, *AICD* amyloid precursor protein intracellular domain, *BACE* b-site amyloid precursor protein cleaving enzyme, *GGA3* Golgi-associated, gamma adaptin ear-containing, ARF-binding protein 3, *p-tau* phosphorylated-tau; sAPPb, soluble amyloid precursor protein b, *VA* volatile anesthetics

An early in vitro study on pheochromacytoma cells confirmed that inhalational anesthetics isoflurane and halothane induced dose- and time-dependent Ab42 oligomerization, even at clinically relevant concentrations, and 1-2MAC of isoflurane or halothane significantly potentiated Ab-induced cytotoxicity in pheochromacytoma [114]. Using neuroglioma H4 cells stably transfected with full-length APP, Xie et al. further demonstrated that 2% isoflurane for 6 h reduced C-terminal precursor protein while it increased Ab40 and Ab42 release, that suggested isoflurane enhanced pathogenic APP cleavage by γ-secretase. Moreover, isoflurane-induced caspase-3 activation to indicate apoptosis, and such effect could not be attenuated by reducing Ab in the cellular environment by Ab-neutralizing antibody or γ-secretase inhibitor; this suggests that isoflurane-induced apoptosis might be independent of Ab accumulation [115]. The relationship between caspase and amyloid-beta was further explored in vivo, wherein 2 h 1.4% of isoflurane increased caspase 3 activation in mouse brain from 6 to 12 h after exposure, which preceded upregulation of b-site APP-cleaving enzyme (BACE, or β-secretase) and Ab at 12–24 h post-exposure. Isoflurane also downregulated Golgi-associated, gamma adaptin ear-containing, ARF-binding protein 3 (GGA-3), an enzyme that degrades BACE and is inhibited by active, cleaved caspase 3. Taken together, such findings may suggest that isoflurane-induced caspase 3 activation could lead to BACE stabilization and promote Ab formation. Conversely, the study also showed that Ab aggregation inhibitor (iAβ5 or clioquinol) reduced caspase 3 activation, to suggest reciprocal regulation of this pathway [116, 117]. A similar in vivo study reported that 2 h 2.5% sevoflurane exposure also elicited the time-specific events of caspase activation, BACE upregulation and Ab production. The study also demonstrated that pan-caspase inhibitor Z-VAD reversed sevoflurane-induced Ab synthesis, to strengthen the hypothesis that caspase 3 activation could be upstream of amyloid processing [118]. Nuclear magnetic resonance studies suggested that some anesthetics could directly interact with Ab to favor oligomerization. Specifically, it was demonstrated that isoflurane and desflurane with their smaller molecular size could interact with critical amino acid residues G29, A30 and I31, which are located within the loop region connecting two Ab helices, and cause chemical shifts in these residues to promote Ab oligomerization at clinically relevant concentrations [119]. Propofol with its larger molecular size was not found to interact with said residues and enhance oligomerization at clinical concentration, yet a positive observation was made at very high propofol concentration [120] (Fig. 4).

Anesthetics have also been shown to promote tau hyperphosphorylation, another hallmark pathology of Alzheimer’s disease. Sevoflurane [121] and propofol [122] were found to induce dose-dependent, transient tau hyperphosphorylation in wildtype mouse brain, with repeated exposure resulting in persistent hippocampal hyperphosphorylation and significant impairment to spatial memory, as assessed by Morris Water Maze test. Anesthetic adjuvant dexmedetomidine also led to reversible tau phosphorylation in WT mouse hippocampus for up to 6 h, yet impairment to spatial memory persisted to 1 week following treatment [123]. In tri-transgenic AD mice harboring APP, presenillin 1 and tau mutations, isoflurane or halothane exposure significantly increased tau phosphorylation in the hippocampal CA1 region, but both anesthetics were not associated with cognitive decline, with halothane even improving memory and learning [113]. In contrast, Li et al. reported that isoflurane-induced hippocampal tau hyperphosphorylation in APP695 mice was accompanied by decline in spatial memory performance [109]. Isoflurane also increased tau phosphorylation in brain tissues and neuronal culture from APP/PS1 transgenic mice, and tau hyperphosphorylation could be attenuated by caspase inhibitor or Aβ generation inhibitor, to suggest that caspase activation and Aβ accumulation could be upstream of tau phosphorylation [124] (Fig. 4).

Clinical studies have also observed changes in cerebral spinal fluid levels of Ab and tau protein in patients following surgery plus general anesthesia. In this regard, CSF total-tau, phosphor-tau, Ab (1–40 or 1–42 form), total-tau/Ab ratio and phosphor-tau/Ab ratio have been some of the most widely used biomarkers aiding the diagnosis of Alzheimer’s disease and dementia, and high specificity and sensitivity can be achieved by combining different biomarkers (e.g., phosphor-tau/Ab ratio plus total tau) [125, 126]. In patients who have undergone endoscopic nasal surgery, GA with sevoflurane or propofol increased CSF total-tau and phosphorylated-tau181P for 48 h to a similar extent without altering CSF Ab1-42 level [127]. A randomized study also reported that the threefold increase in CSF tau protein level and tau/Ab ratio is independent of anesthetic type (isoflurane vs. propofol) 24 h following neurosurgery/otolaryngeal surgery, with minimal effect on CSF Ab level [128]. Moreover, in patients receiving lower extremity or lower abdominal surgery, compared to spinal anesthesia alone, combined anesthesia with isoflurane increased CSF level of Ab40 at 24 h, whereas when combined with desflurane CSF, Ab42 decreased 2 h after surgery; the three modes of anesthesia did not differ in their effects on CSF tau protein [129]. In conclusion, these preliminary clinical studies partially concur with observations from in vitro and animal studies, however, their relatively small sample size and different surgery/anesthesia protocols limit further interpretation of the findings.

Current clinical evidences on this subject are far from conclusive. A meta-analysis on 15 case–control studies reported that GA exposure, single or cumulative, is not associated with higher risk of AD [130] compared to no-surgery/anesthesia control or regional anesthesia; a prospective cohort study similarly concluded that GA does not significantly increase dementia/AD incidence during a 7-year follow-up [131]. In contrast, a nationwide case–control study reported that subjects receiving surgery and general anesthesia are at higher risk of developing dementia, in particular with multiple surgery/anesthesia challenge, when compared to no-surgery/anesthesia controls [132]. Consistent with such, a cohort study concluded that surgery plus anesthesia is associated with increased incidence of dementia and reduced time interval to dementia diagnosis, regardless of the mode of anesthesia received (general or regional) [133].

## Conclusion

The developing and aging brain may be vulnerable to anesthesia. An important mechanism for anesthesia-induced developmental neurotoxicity is widespread neuroapoptosis, whereby an early exposure to anesthesia causes long-lasting impairments in neuronal communication and faulty formation of neuronal circuitries. Exposure to anesthesia to the aged brain can be a risk of the long-lasting impairments of cognitive function. However, the neuroprotective property of general anesthetics in brain injury is also increasingly recognized. That is to say, one should bear in mind the “Ying and Yang” balance of general anesthetics in daily clinical practice. Once this is implemented well, patients will be benefit from “precision” anesthesia. In addition, one should also consider the detrimental effects of trauma induced by surgery on vital organs; in particular, systemic inflammatory responses following surgery can cause various organ injury/dysfunction including cognitive impairment [134]. Therefore, how the perioperative team including anesthetists, surgeons and intensivists should work together in an optimal manner is important for the best benefits of our patients.
